# Grapes

**Published:** 1874-09

**Authors:** 


### GRAPES.

Every farm-house may have fresh grapes from the vine all winter up to May, and as they are exceedingly grateful to the sick, in all forms of fevers, it is worth a little trouble to have them at hand. Leave the grapes on the vine out-doors as long as possible, without freezing, for the longer they thus remain the sweeter they become; the first frost, unless very severe, will not injure them. The bunch, in cutting, is not detached from the stalk or cane, but the latter is cut so that the cluster is attached to it, after cutting, two or three knots or joints below the cluster, and two above. The upper end is then covered with wax, to prevent the evaporation of the fluids contained within the pores of the wood. All grapes, not absolutely healthy, are carefully removed from the cluster, after which the lower end of the stock is thrust through the hole in a perforated cork, and down into a bottle filled with water. In the water is a little wood-charcoal, which prevents it from becoming impure. The cork is crowded into the mouth of the bottle as closely as possible, and then covered with sealing wax around the stalk, so as to close the bottle, water and air tight. The bottles aie then placed upon tables or shelves in a dry chamber, in which the temperature never falls below the freezing point. The bottles are supported in any convenient way, so as to prevent their being tipped over by the weight of the clusters, and are placed at such intervals that the branches do not come in contact with each other. The bunches must be, from time to time, carefully examined, and such single grapes as show symptoms of spoiling must be removed.

